# Chitosan-Crosslinked Low Molecular Weight PEI-Conjugated Iron Oxide Nanoparticle for Safe and Effective DNA Delivery to Breast Cancer Cells

**DOI:** 10.3390/nano12040584

**Published:** 2022-02-09

**Authors:** Guanyou Lin, Jianxi Huang, Mengyuan Zhang, Shanshan Chen, Miqin Zhang

**Affiliations:** Department of Materials Science and Engineering, University of Washington, Seattle, WA 98195, USA; linguany@uw.edu (G.L.); jianxih@uw.edu (J.H.); zhangm28@uw.edu (M.Z.); schen268@jh.edu (S.C.)

**Keywords:** iron oxide nanoparticles, gene therapy, breast cancer

## Abstract

Breast cancer has attracted tremendous research interest in treatment development as one of the major threats to public health. The use of non-viral carriers for therapeutic DNA delivery has shown promise in treating various cancer types, including breast cancer, due to their high DNA loading capacity, high cell transfection efficiency, and design versatility. However, cytotoxicity and large sizes of non-viral DNA carriers often raise safety concerns and hinder their applications in the clinic. Here we report the development of a novel nanoparticle formulation (termed NP-Chi-xPEI) that can safely and effectively deliver DNA into breast cancer cells for successful transfection. The nanoparticle is composed of an iron oxide core coated with low molecular weight (800 Da) polyethyleneimine crosslinked with chitosan via biodegradable disulfide bonds. The NP-Chi-xPEI can condense DNA into a small nanoparticle with the overall size of less than 100 nm and offer full DNA protection. Its biodegradable coating of small-molecular weight xPEI and mildly positive surface charge confer extra biocompatibility. NP-Chi-xPEI-mediated DNA delivery was shown to achieve high transfection efficiency across multiple breast cancer cell lines with significantly lower cytotoxicity as compared to the commercial transfection agent Lipofectamine 3000. With demonstrated favorable physicochemical properties and functionality, NP-Chi-xPEI may serve as a reliable vehicle to deliver DNA to breast cancer cells.

## 1. Introduction

The ability to modulate genetic behaviors of cells opens up a wide array of possibilities in treating diseases such as cancer. As a major threat to public health, breast cancer is the most common cancer in the female population with approximately 2.3 million new cases and 685,000 deaths worldwide in 2020 alone [1]. Gene therapy enabled by plasmid DNA or mRNA transfection has been proven to be efficacious for treating breast cancers in recent studies [2,3,4]. Despite the recent fervor for mRNA therapy, DNA remains as a promising therapeutic option for anticancer gene therapy because (1) DNA is more resistant to heat and enzymatic degradation than mRNA [5] and (2) mRNA is more immunogenic than DNA and can cause unwanted inflammation and autoimmune responses [6,7]. Nonetheless, successful DNA transfection in cancer cells is not without challenges, which include cellular membrane penetration and timely DNA release. Judicious gene carrier designs are needed to overcome these challenges. Non-viral gene carriers have captured much of the spotlight in gene delivery research owing to their versatile designs, facile production, and high loading capacity [8]. Moreover, the safety concerns of viral vector-related immunogenicity [9] and mutagenesis [10] also led to the active development of non-viral carriers.

In successful non-viral DNA carriers, which are mostly composed of cationic polymers and lipids, several critical factors are well-balanced. The presence of sufficient cationic moieties is required to tightly condense DNA for its protection from nucleases and facile cell uptake. Meanwhile, condensed DNA also needs to be unwrapped and enter cell nuclei before the transcription can occur. In addition to the intricate balance between DNA condensation and release, the relationship between transfection efficiency and cytotoxicity also needs to be balanced. Insufficient cationic moieties cannot effectively condense DNA while the over-abundance of cationic moieties could damage cells by destabilizing cellular membranes [11,12] and releasing radical oxygen species [13]. The optimal size and zeta potential range of non-viral gene carriers are between 50 and 100 nm and around 10 mV, respectively [14]. Serum stability is also critical for the non-viral DNA carrier’s performance. If the DNA carrier’s surface chemistry is not properly tuned, serum proteins could form thick layers on the DNA complex and significantly increase its size and hinder the cell uptake process downstream. Therefore, fine-tuning the chemical composition and in turn the physicochemical property of a non-viral DNA carrier to find the balance between various critical factors is crucial.

Known for their superior transfection efficiency but also their pronounced cytotoxicity [15], cationic lipids became the attractive subjects for chemical modifications aiming to reduce their toxicity while retaining their transfection prowess. However, cationic lipids are rarely a stand-alone DNA condensing agent due to the limited reactive sites for modifications in their headgroups (usually less than 10) [16]. Hence, cationic lipids are usually accompanied by other helper lipids, such as phospholipids and cholesterol-derivatives, to form lipoplexes with DNA. Nonetheless, a multi-component lipoplex system would require tremendous efforts in optimizing the relationship between each lipid component before satisfactory DNA delivery results can be achieved. As another gold standard in non-viral gene delivery, high molecular weight (HMW) PEI also faces the quandary of being highly efficient in transfection and toxic at the same time [17,18,19]. Fortunately, PEI possesses abundant reactive amine groups for versatile functionalization and has the potential to be decorated as a stand-alone DNA condensing agent [20,21,22,23]. Interestingly, it has also been found that PEI can promote the nuclear entry of DNA for transcription while lipid-bound DNA is not transcription-active [24,25]. The toxicity related to high molecular weight PEI [26,27] can be greatly alleviated by crosslinking low molecular weight PEI via biodegradable linkages such as disulfides [28,29,30,31,32,33]. Nevertheless, most of the polyplexes formed by these crosslinked PEI and DNA are hundreds of nm in size; large cationic polyplexes have more difficulty entering cells and can inflict cell damage by the sheer amount of positive charges they carry if they are not rapidly degraded into nontoxic small segments after cellular entry [34,35].

Iron oxide nanoparticles (IONPs) have shown promises as a carrier platform for DNA delivery due to their biocompatibility, tunable surface chemistry for modifications, and exploitable magnetic properties. IONPs are commonly coated with high molecular weight PEI (e.g., 10 kDa and 25 kDa PEI) for effective DNA condensation and intracellular trafficking. However, they will inevitably inherit some toxicity from non-degradable large PEI [36,37,38]. Even though the rigid inorganic core of an IONP can provide structural support for DNA complex to render its size and shape more controllable [39], it is not uncommon that IONP-based DNA delivery systems could still be hundreds of nanometers in size. It has also been noted that the presence of serum could significantly hinder the transfection of IONP-based DNA complexes [40,41]. If an IONP-based DNA carrier can integrate the conducive features of small size, serum stability, and biodegradable cationic coating all at once, its transfection performance will likely be significantly enhanced. Herein, we report the development of an ultrasmall, serum-stable and biodegradable IONP-based nanocarrier for safe and effective DNA delivery to various types of breast cancer cells. The nanoparticle, termed NP-Chi-xPEI, was constructed by first crosslinking low molecular weight branched PEI (800 Da) molecules with linear chitosan (MW 3.9 kDa, deacetylation degree 90%) chains to form chitosan-PEI crosslinked polymer (Chi-xPEI), followed by conjugating Chi-xPEI onto a 10-nm superparamagnetic iron oxide nanoparticle (NP) to form NP-Chi-xPEI. Low molecular weight branched PEI was chosen for its favorable biocompatibility and its branched structure for DNA condensing capability [42]. A biodegradable disulfide-containing homo-bifunctional crosslinker dithiodipropionic acid (DTDPA) was used to crosslink PEI so that the crosslinked polymer can be degraded into non-toxic fragments in cytoplasm after DNA delivery [43]. Chitosan, a primary amine-rich linear polysaccharide, serves as the binding template for PEI molecules to boost the crosslinking efficiency as well as enhance the biocompatibility of the crosslinked polymer [44]. The 10-nm NP provides a robust inorganic platform for crosslinked polymers to reside on and in turn contribute to a more controllable ultrasmall size profile for the entire DNA carrier system. With its high in vitro transfection efficiency and minimal cytotoxicity demonstrated on multiple breast cancer cells, NP-Chi-xPEI could be a potent DNA carrier for breast cancer gene therapy.

## 2. Materials and Methods

### 2.1. Materials

Plasmid pDsRed-MAX-N1 was purchased from Addgene (Watertown, MA, USA). DH5–α competent *E. coli* was purchased from New England Biolabs Inc (Ipswich, MA, USA). Plasmid Giga Kit was purchased from Qiagen (Germantown, MD, USA). Chitosan was purchased from Acmey Industrial (Shanghai, China). DAPI, Lipofectamine 3000, 2-Iminothiolane (Traut’s reagent), Succinimidyl Iodoacetate (SIA), Ultrapure Agarose, antibiotic-antimycotic, Tryple Express Enzyme solution, RPMI 1640 and DMEM cell culture medium were purchased from Invitrogen (Carlsbad, CA, USA). PD10 desalting columns, Sephacryl S-200 resin, and HyClone characterized fetal bovine serum (FBS) were purchased from GE Healthcare Life Sciences (Pittsburgh, PA, USA). NHS-Cy5 and NHS-AF488 were purchased from Lumiprobe Corp (Hunt Valley, MD, USA). Label IT Tracker Intracellular Nucleic Acid Localization Kits were purchased from Mirus Bio (Madison, WI, USA). SpectraPOR7 dialysis membrane was purchased from Repligen Corp (Waltham, MA, USA). All other chemicals were purchased from Sigma-Aldrich (St Louis, MO, USA).

### 2.2. Plasmid DNA Preparation

The pDsRed-MAX-N1 plasmid was propagated in DH5-α *E. coli* competent cells and purified using the Plasmid Giga Kit following the manufacturer’s procedures. Purified pDsRed-MAX-N1 was dissolved in ultrapure water at 1 mg/mL and stored at −20 °C.

### 2.3. Nanoparticle and Polymer Synthesis

The synthesis procedure of iron oxide nanoparticles coated with primary amine functionalized PEG (NP) has been reported previously [45]. The Chi-xPEI polymer was produced by crosslinking chitosan (MW 3.9 kDa, deacetylation degree 90%) with low molecular weight branched PEI (MW 800 Da) via a disulfide-containing homo-bifunctional crosslinker dithiodipropionic acid (DTDPA). The molar ratio between DTDPA, PEI, and chitosan was 8.00:5.00:0.25. Specifically, the terminal carboxylic acid groups on DTDPA (40 mg/mL in DMSO) were first activated by 1-Ethyl-3-(3-dimethylaminopropyl) carbodiimide/N-hydroxysuccinimide (EDC/NHS) chemistry with the DTDPA:EDC:NHS molar ratio of 1.0:1.5:1.5 in DMSO for 3 h with vigorous stirring at room temperature. PEI and chitosan were dissolved in DMSO at 200 mg/mL and 15 mg/mL respectively. 800 Da PEI and chitosan solution were added to the DTDPA solution drop-wisely and the mixture was vigorously stirred at room temperature for 16 h. An equal volume of Milli-Q water was added to the mixture DMSO solution, and the resultant solution was dialyzed against Milli-Q water for 2 days using 25k MWCO SpectraPOR7 dialysis membrane. The dialyzed solution was then freeze-dried, re-dissolved in Milli-Q water at 200 mg/mL of Chi-xPEI and stored at 4 °C.

### 2.4. Quantification of Iron [Fe] Concentration in NP by Ferrozine Assay

The ferrozine solution was prepared by dissolving 1.761 g ascorbic acid, 1.927 g ammonium acetate, 0.032 g ferrozine, and 0.0135 g neocuproine in 10 mL of DI H_2_O. A volume of 1000 ppm Fe stock solution was diluted to 1 ppm, 2 ppm, and 4 ppm with 10 mM HCl as Fe standards. A volume of 10 mM HCl was used as a 0 ppm Fe control. To prepare the NP sample, 5 μL of NP solution was dissolved in 45 μL of concentrated HCl, followed by 50× dilution in DI H_2_O. A volume of 6 μL of the diluted NP solution was mixed with 694 μL of DI H_2_O and 90 μL of ferrozine solution. A volume of 300 μL of each 0 ppm, 1 ppm, 2 ppm, and 4 ppm Fe standards were then mixed with 400 μL of DI H_2_O and 90 μL of ferrozine solution. After 10 min, the 562 nm absorbance of the samples was measured with a SpectraMax i3 multimode microplate reader (Molecular Devices, Sunnyvale, CA, USA). The iron concentration of NP is denoted as [Fe].

### 2.5. Conjugation of Chi-xPEI onto NP

An amount of 1 mg [Fe] NPs were reacted with 0.1 mg 2-iminothiolane (Traut’s Reagent) for 1 h in thiolation buffer (0.1 M sodium bicarbonate, pH 8.0, 5 mM EDTA) before removing unreacted Traut’s Reagent using a PD-10 column equilibrated with thiolation buffer. Concurrently, 40 mg Chi-xPEI was reacted with 1.35 mg succinimidyl iodoacetate in thiolation buffer. The modified Chi-xPEI was then added to NP-Traut’s solution and the resultant solution was gently rocked for 2 h before placed at 4 °C for 16 h to drive the reaction to completion. Unreacted Chi-xPEI was removed through size exclusion chromatography using S-200 Sephacryl resin equilibrated with 20 mM HEPES buffer (pH 7.4).

### 2.6. NMR Analysis

The polymer samples were prepared by dissolving polymer and TSP in D_2_O and NMR spectra were obtained using a Bruker Avance 300 spectrometer (Bruker, Billerica, MA, USA) operating at 300.13 MHz (^1^H) and 298 K (number of scans = 64, acquisition time = 3.9 s, delay (D1) = 2 s).

### 2.7. FTIR Analysis

The FTIR spectra were obtained using a Nicolet 6700 spectrometer (Thermo Scientific Inc., Waltham, MA, USA). The spectra were obtained at 4 cm^−1^ resolution and the signal was averaged over 64 scans. The samples were pressed into a pellet with KBr for analysis.

### 2.8. NP-Chi-xPEI-DNA Complex Formation

The NP-Chi-xPEI and DNA (pDsRed-MAX-N1) were mixed in 20 mM HEPES buffer (pH 7.4) at NP-Chi-xPEI [Fe]:DNA wt/wt ratios of 1:1, 5:1, and 10:1. NP-Chi-xPEI-DNA solutions were incubated for at least 10 min with gentle rocking to allow the formation of DNA complexes.

### 2.9. TEM Imaging

The TEM samples were prepared by the addition of 5 μL of NP, NP-Chi-xPEI, or NP-Chi-xPEI-DNA solution to a Formvar/carbon-coated 300-mesh copper grid (Ted Pella, Inc., Redding, CA, USA) and allowed to air dry. The TEM images were acquired on a Tecnai G2 F20 electron microscope (FEI, Hillsboro, OR, USA) operating at a voltage of 200 kV.

### 2.10. Hydrodynamic Size and Zeta Potential Measurements

The hydrodynamic sizes and zeta potentials of NP-Chi-xPEI and NP-Chi-xPEI-DNA complexes were obtained using a Zetasizer Nano-ZS (Malvern Instruments, Worcestershire, UK). The samples were analyzed in 20 mM HEPES buffer at room temperature. For the serum stability study, the NP-Chi-xPEI [Fe]:DNA wt/wt ratios of 10:1 solution was mixed with RPMI-1640 cell culture medium (supplemented with 10% FBS and 1% antibiotic-antimycotic) to achieve 10% *v*/*v* of NP-Chi-xPEI in medium and placed in 37 °C water bath for the duration of experiment.

### 2.11. Cell Culture

The SKBR3 and MCF7 cells were cultured in DMEM supplemented with 10% FBS and 1% antibiotic-antimycotic. 4T1 cells were cultured in RPMI-1640 supplemented with 10% FBS and 1% antibiotic-antimycotic. The cultures were maintained at 37 °C in a humidified incubator with 5% CO_2_.

### 2.12. Fluorophore Labeling of NP-Chi-xPEI and DNA

A volume of 2 μL of NHS-AF488 fluorophore (250 mM in DMSO) was mixed with 1 mg of [Fe] NP-Chi-xPEI and the mixture was gently rocked for 30 min at room temperature. The unreacted NHS-AF488 molecules were removed by PD-10 desalting columns equilibrated with 20 mM HEPES buffer (pH 7.4). The DNA was labeled with Cy5 following the manufacturer’s protocol of the Label IT Tracker Intracellular Nucleic Acid Localization Kits.

### 2.13. Cellular Uptake and Intracellular Plasmid DNA Release Studies

The SKBR3, MCF7, and 4T1 cells were seeded at 15,000, 15,000, 8000 cells per well in 24-well plates, respectively, and incubated for 16 h. For the cellular uptake study, NP-Chi-xPEI was complexed with Cy5-labeled DNA at 10:1 wt/wt [Fe] NP-Chi-xPEI:DNA ratio before adding to cells at 1 μg/mL DNA concentration. For the DNA release study, NP-Chi-xPEI and DNA were labeled with NHS-AF488 and Cy5, respectively, before forming a complex at 10:1 wt/wt [Fe] NP-Chi-xPEI:DNA ratio. The NHS-AF488 and Cy5-dually labeled NP-Chi-xPEI-DNA complexes were added to cells at 1 μg/mL of DNA concentration. Fluorescently labeled NP-Chi-xPEI-DNA complexes were incubated with cells for 24 h before imaging with a Nikon TE300 inverted fluorescent microscope (Nikon, Tokyo, Japan) in both studies.

### 2.14. Cell Transfections

The SKBR3, MCF7, and 4T1 cells were seeded at 15,000, 15,000, 8000 cells per well in 48-well plates, respectively, for 16 h. The NP-Chi-xPEI-DNA complexes prepared at a 10:1 wt/wt ratio of [Fe] NP-Chi-xPEI:DNA were added to 200 μL of fully supplemented culture media to give a final DNA concentration of 2 μg/mL in each well. The cells were incubated with complexes for 48 h and the cell culture media were replenished after 24 h. Transfections using the commercial agent, Lipofectamine 3000, was performed following the manufacturer’s protocol. A DNA concentration of 2 μg/mL was used for all the transfection agents for the transfection study. The cells were imaged 72 h post-transfection with a Nikon TE300 inverted fluorescent microscope (Nikon, Tokyo, Japan).

### 2.15. Cell Viability Studies

The SKBR3, MCF7, and 4T1 cells were seeded at 7500, 7500, 4000 cells per well in 96-well plates, respectively, and incubated for 24 h. The cells were then incubated with NP-Chi-xPEI-DNA at 10:1 wt:wt ratio of [Fe] NP-Chi-xPEI:DNA or Lipofectamine-DNA, all at DNA concentrations of 0, 0.25, 0.5, 1, 2, 4 μg/mL. The cells were treated for 24 h before the cell viability was determined using the Alamar Blue assay. The fluorescent signal readout was obtained by a SpectraMax i3 microplate reader (Molecular Devices, Sunnyvale, CA, USA) with 550 nm excitation and 590 nm emission. The fluorescence intensities of all the treatment groups were normalized so that the viability of the untreated cell group was 100%.

### 2.16. Statistical Analysis

The results are presented as mean values ± standard error of the mean. The statistical differences were determined by two-sided Student’s *t*-test. The values were considered statistically significant at *p* < 0.05.

## 3. Results and Discussion

### 3.1. Synthesis and Structural Validation of NP-Chi-xPEI

**Figure 1:**
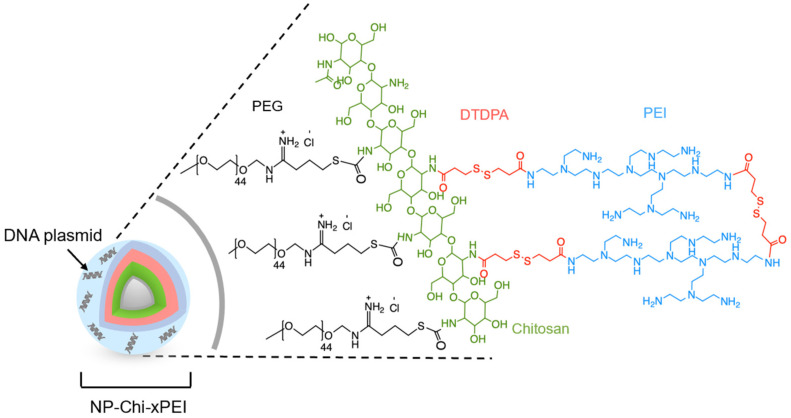
Schematic representation of NP-Chi-xPEI complexed with DNA with the zoom-in details of the structure of the Chi-xPEI polymer conjugated onto the surface of NP.

**Figure 2:**
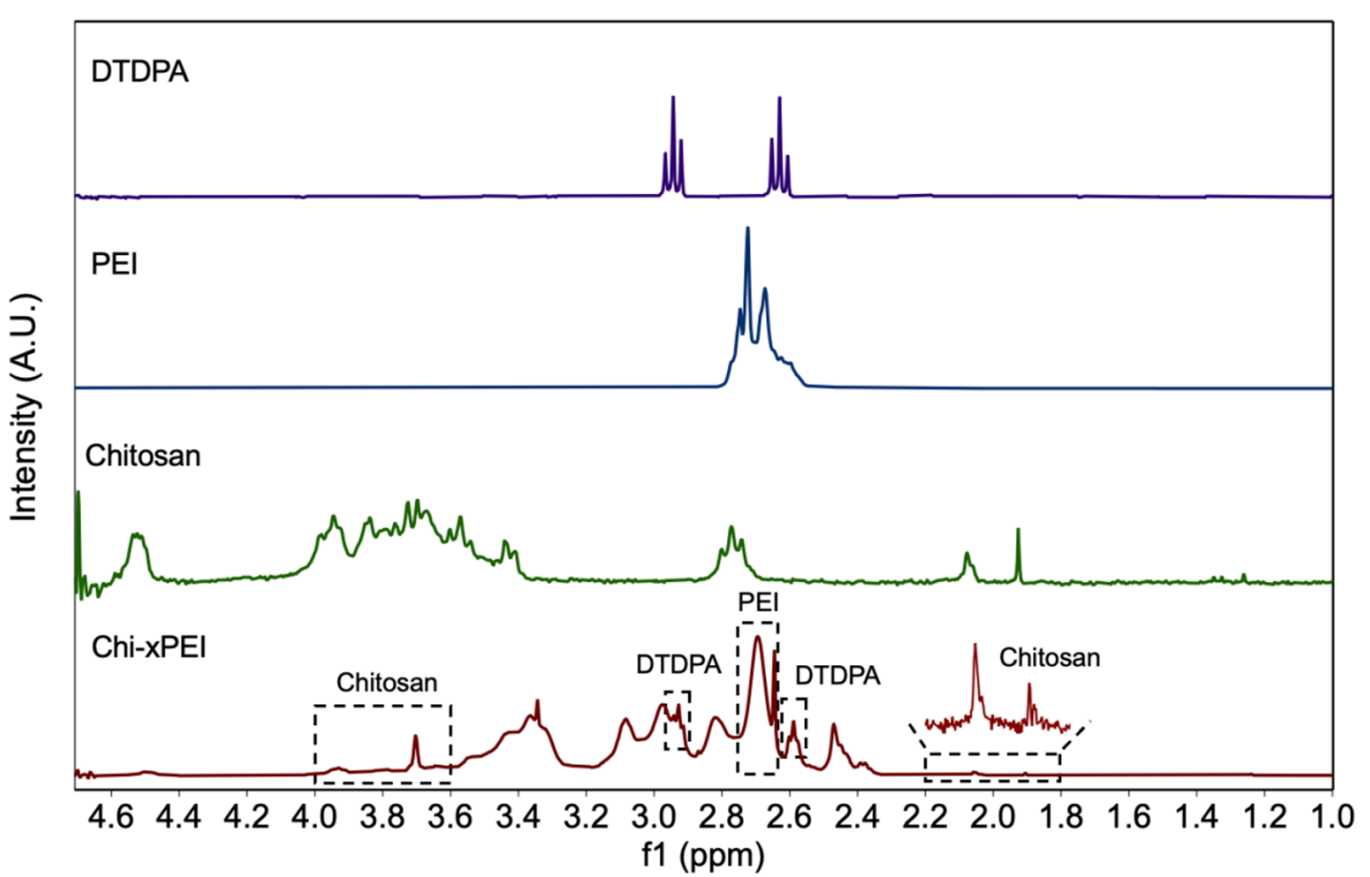
^1^H NMR spectra of DTDPA, chitosan, PEI, and Chi-xPEI. Dashed boxes on the Chi-xPEI spectrum indicate the presence of characteristic peak patterns from its constituents.

**Figure 3:**
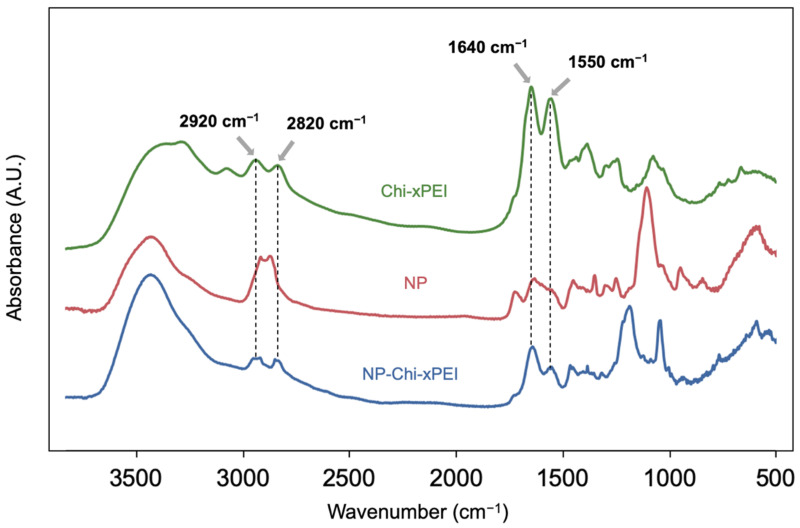
FTIR absorbance spectra of Chi-xPEI, NP, and NP-Chi-xPEI.

The 10 nm-diameter iron oxide nanoparticles coated with primary amine functionalized PEG were prepared as previously described [45] (herein termed as NP). The coating polymer Chi-xPEI was produced by crosslinking chitosan (MW 3.9 kDa) with low molecular weight branched PEI (MW 800 Da) via a disulfide-containing homobifunctional crosslinker dithiodipropionic acid (DTDPA). The Chi-xPEI was then covalently conjugated onto NP via succinimidyl iodoacetate (SIA)/2-iminothiolane (Traut’s reagent) chemistry to confer positive charges onto the NP surface for condensing DNA. The resultant surface structure of NP-Chi-xPEI is shown in Figure 1. To confirm the successful crosslinking of chitosan and PEI via DTDPA, ^1^H NMR spectra of Chi-xPEI and its constituents including chitosan, PEI, and DTDPA were acquired (Figure 2). The NMR spectrum of Chi-xPEI contains the characteristic peaks from all the crosslinking constituents, as evidenced by the double triplet peaks in the 2.5–3.0 ppm region from DTDPA, the broad convoluted peaks in the 3.5–4.0 ppm region and 1.8–2.2 ppm from chitosan and the strong absorbance peak pattern around 2.7 ppm from PEI. The FTIR was then used to validate the conjugation of Chi-xPEI onto NP. The FTIR absorbance spectra of Chi-xPEI, NP, and NP-Chi-xPEI were acquired and compared (Figure 3). Different from the NP’s FTIR spectrum, the spectrum of NP-Chi-xPEI inherits peaks at 2920 cm^−1^, 2820 cm^−1^, 1640 cm^−1^, and 1550 cm^−1^ from Chi-xPEI. Specifically, the stretching of C-H bonds accounts for the absorbance peaks at 2920 cm^−1^ and 2820 cm^−1^, while the bending of N-H bonds corresponds to peaks at 1640 cm^−1^ and 1550 cm^−1^ [46]. The presence of the featuring FTIR peaks of Chi-xPEI in NP-Chi-xPEI’s spectrum but not in NP’s spectrum corroborates the successful conjugation of Chi-xPEI onto NP.

### 3.2. Physicochemical Properties

**Figure 4:**
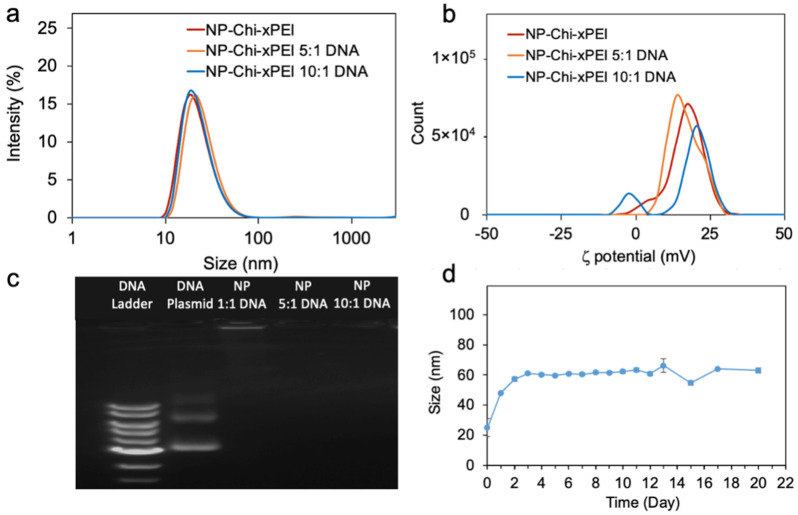
Physicochemical properties of NP-Chi-xPEI-DNA complex. (**a**) Hydrodynamic size distributions of NP-Chi-xPEI, NP-Chi-xPEI 5:1 DNA, and NP-Chi-xPEI 10:1 DNA. (**b**) Zeta potentials of NP-Chi-xPEI, NP-Chi-xPEI 5:1 DNA, and NP-Chi-xPEI 10:1 DNA. (**c**) Gel electrophoresis image of NP-Chi-xPEI, NP-Chi-xPEI 5:1 DNA, and NP-Chi-xPEI 10:1 DNA. Here, NP-Chi-xPEI is abbreviated as NP. (**d**) Serum stability of NP-Chi-xPEI 10:1 DNA in RPMI 1640 cell culture medium supplemented with 10% FBS assessed by hydrodynamic size measurements over a period of 20 days.

The appropriate size and surface charge are essential for non-viral gene carriers to promote cellular uptake and maximize transfection efficiency. It was shown that nanoparticles with hydrodynamic size near to or smaller than 100 nm can be internalized most efficiently by various cell types in vitro [47]. Moreover, nanoparticles of larger than 100 nm will be easily taken up by macrophage cells of the reticuloendothelial system (RES) in liver and spleen, while those smaller than 10 nm will be filtered out by the kidneys in vivo [48]. The compact sizes of NP-Chi-xPEI (before DNA complexation), NP-Chi-xPEI [Fe] 5:1 DNA, and NP-Chi-xPEI [Fe] 10:1 DNA complexes, whose hydrodynamic diameters are 31.33 nm, 53.24 nm, and 45.79 nm, respectively (Figure 4a), not only facilitate the cell internalization of these complexes but could also be favorable for long-term in vivo trafficking. Although positive surface charge is important for nanoparticles’ cellular internalization as it can facilitate nanoparticles’ attachment to anionic cell plasma membrane and prevent nanoparticle agglomeration via charge repulsion, too high of a positive charge may also increase the risk of organelle damages [49]. It has been shown that effective nanoparticle DNA carriers typically possess a ζ potential of near 20 mV [36,50,51]. The ζ potential of NP-Chi-xPEI, NP-Chi-xPEI [Fe] 5:1 DNA, and NP-Chi-xPEI [Fe] 10:1 DNA are 11.8 mV, 17.0 mV, and 18.3 mV, respectively (Figure 4b), conforming that NP-Chi-xPEI retains an appropriate amount of positive charge for transfection after DNA condensation. When compared to other recently developed non-viral DNA carriers, the hydrodynamic size and zeta potential profiles of NP-Chi-xPEI-DNA is superior to most of the biodegradable polymeric PEI-DNA polyplexes (Table 1) and IONP-based DNA carriers (Table 2) as it is more compact in size while retaining sufficient charge. This could be mostly attributed to the size-conservation effect of the small 10 nm NP core which provides a solid support with a well-defined shape for Chi-xPEI conjugation. The NP’s contribution to small DNA complex size is apparent when comparing the hydrodynamic size and zeta potential profiles of NP-Chi-xPEI-DNA to that of Chi-xPEI-DNA without NP (Figure S1). The Chi-xPEI-DNA’s hydrodynamic size was significantly larger than that of NP-Chi-xPEI-DNA (223 nm vs. 45.8 nm) but with similar zeta potential (18.7 mV vs. 18.3 mV).

A desirable feature of a DNA nanocarrier is the ability to protect DNA from degradation by nucleases and destructive enzymes within the endolysosome in the target cell before DNA’s endosomal escape and transportation to the cell nucleus. The DNA protection and condensation were also examined through gel retardation assay (Figure 4c). At both 5:1 and 10:1 NP-Chi-xPEI [Fe]:DNA wt/wt ratios, NP-Chi-xPEI was able to protect and condense DNA effectively, as shown by the absence of discernible free DNA bands. A small amount of DNA was observed only in the loading well at 1:1 NP-Chi-xPEI [Fe]:DNA wt/wt ratio, indicating the DNA was mostly condensed but not fully protected at such ratio. Compared to NP-Chi-xPEI-DNA, Chi-xPEI (with no NP) was unable to fully protect DNA as shown by the DNA signal clearly observed in the loading well even at 40:1 of Chi-xPEI to DNA wt/wt ratio (Figure S2). This phenomenon could be explained by the fact that NP-Chi-xPEI could condense DNA into a more compact size and hence provide better protection than Chi-xPEI does. Given that the NP-Chi-xPEI-DNA complex at [Fe] 10:1 DNA wt/wt ratio not only could fully protect DNA but also possesses small size and sufficient positive charges, NP-Chi-xPEI [Fe] 10:1 DNA (abbreviated as NP-Chi-xPEI-DNA henceforth) was selected for further studies.

To determine the size stability of the nanoparticles in serum, NP-Chi-xPEI-DNA was placed into cell culture medium (RPMI 1640 + 10% FBS) and its hydrodynamic diameter was monitored for 20 days (Figure 4d). The initial size of NP-Chi-xPEI-DNA was around 20 nm at day 0 even though the hydrodynamic size of NP-Chi-xPEI-DNA was shown to be 45.79 nm earlier. This phenomenon could be explained by the fact that the presence of free small serum proteins in the cell culture media at the beginning of the study lowered the average size of the sample. As the free serum proteins started to attach onto NP-Chi-xPEI-DNA’s surface, NP-Chi-xPEI-DNA’s size gradually increased to around 60 nm (similar to its original 45.79 nm hydrodynamic size) and reached stable equilibrium within 48 h. Most importantly, NP-Chi-xPEI-DNA was able to maintain its size at around 60 nm in cell culture medium until the 20th day, demonstrating its ability to stay unaggregated in serum.

**Figure 5:**
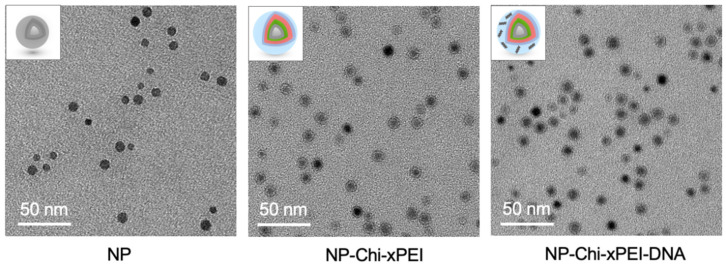
TEM micrographs of NP, NP-Chi-xPEI, and NP-Chi-xPEI-DNA. Insets are structural illustrations of NP, NP-Chi-xPEI and NP-Chi-xPEI-DNA corresponding to TEM micrographs.

TEM was employed to assess the size and shape of NP-Chi-xPEI-DNA. TEM micrographs revealed the spherical shape and mono-dispersed NP and NP-Chi-xPEI with the size of around 10 nm (Figure 5). The planar distance of 0.29 nm in NP crystal lattice, which corresponds to the characteristic {220} planes of Fe_3_O_4_, confirms the composition of magnetite NP core (Figure S3) [57,58,59]. The halo surrounding the NPs in the NP-Chi-xPEI micrograph but absent in the NP micrograph could be attributable to the surface coating of Chi-xPEI on NP. In the NP-Chi-xPEI-DNA micrograph (DNA invisible in the micrograph due to TEM high resolution setting), NP-Chi-xPEIs-DNA were brought to closer vicinity than those in the NP-Chi-xPEI micrograph, indicating the possibility of several NP-Chi-xPEI being electrostatically drawn to a single DNA molecule (Figure 5). This phenomenon could be attributed to the larger hydrodynamic size of NP-Chi-xPEI-DNA than that of NP-Chi-xPEI. The fact that NP-Chi-xPEI was able to stay dispersed after complexed with DNA suggests that DNA can be effectively condensed by individual or only a few NP-Chi-xPEI into compact size without the need to form large DNA complex.

### 3.3. Cellular Uptake and Intracellular Plasmid DNA Release

As a prerequisite for successful transfection, the cellular uptake of NP-Chi-xPEI-DNA was investigated on SKBR3, MCF7, and 4T1 breast cancer cell lines. SKBR3 and MCF7 are both derived from human breast cancer cell lines and have been proven useful preclinical models for screening therapeutic agents that target human epidermal growth factor receptor 2 (HER2) and estrogen receptor-α (ER), respectively [60,61]. The 4T1 cells are murine breast cancer cells and have been utilized to resemble human metastatic triple-negative (negative expression of ER, progesterone receptor (PR), and HER2) breast cancer [62].

**Figure 6:**
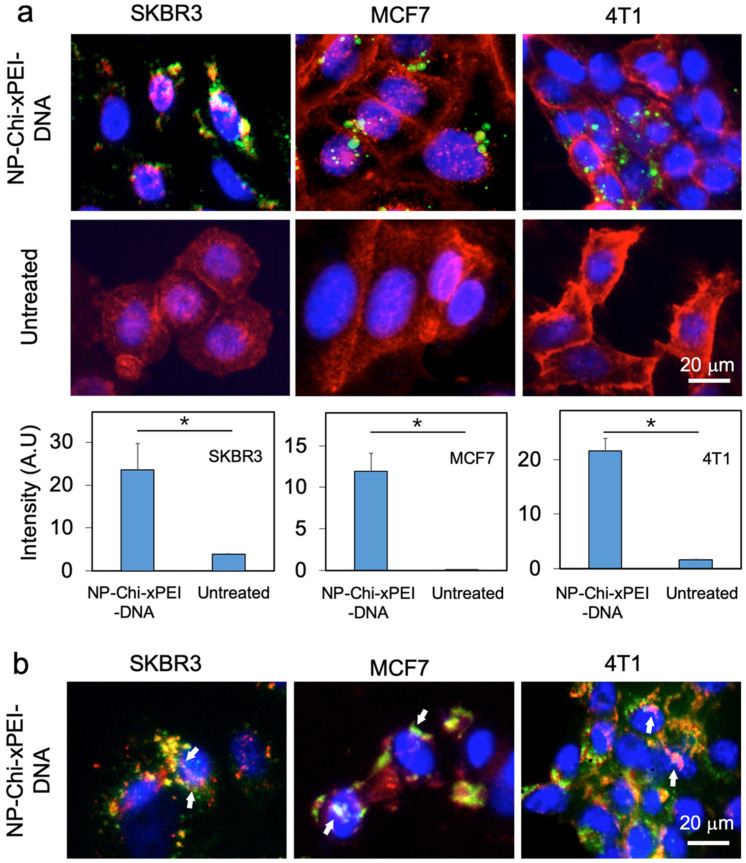
Cellular uptake and intracellular plasmid DNA release by NP-Chi-xPEI. (**a**) Fluorescent images of cellular uptake of NP-Chi-xPEI-DNA. The cell nucleus was stained blue and the cell membrane stained red. The DNA was tagged with Cy5 (green). Quantitation of DNA cellular uptake is presented in bar charts, with * *p* < 0.05. (**b**) Fluorescent images of DNA released from NP-Chi-xPEI-DNA. The cell nucleus was stained blue. NP-Chi-xPEI was tagged with AF488 (red) and plasmid DNA tagged with Cy5 (green). White arrows point at the DNA colocalized within cell nuclei and accompanied by NP-Chi-xPEI in vicinity. For both experiments, NP-Chi-xPEI-DNA was incubated with cells at 1 μg/mL of DNA concentration for 24 h before imaging.

To identify nanoparticles’ intracellular spatial distribution, NP-Chi-xPEI was complexed with Cy5 fluorophores (in green)-tagged plasmid DNA. The three cell lines in 24-well plates were treated with NP-Chi-xPEI-DNA complex at 1 μg/mL DNA concentration for 24 h before their nuclei were stained with DAPI (blue) and plasma membranes with WGA-AF555 (red). It was observed from the images (Figure 6a) that a significant amount of NP-Chi-xPEI-DNA penetrated plasma membrane and arrived in the intracellular spaces of SKBR3, MCF7, and 4T1 cells, indicating the cellular internalization process was efficient for NP-Chi-xPEI-DNA. Even though NP-Chi-xPEI can efficiently ferry DNA across the plasma membrane, it would be difficult to transcribe DNA if DNA remained tightly condensed by NP-Chi-xPEI. Therefore, DNA and NP-Chi-xPEI had been labelled with Cy5 (green) and AF488 (red) respectively before complexation to form dual-labeled NP-Chi-xPEI-DNA to investigate the process of DNA release from NP-Chi-xPEI-DNA. NP-Chi-xPEI-DNA was incubated with three cell lines, respectively, at 1 μg/mL of DNA concentration for 24 h before fluorescent images were acquired. From the images of these three cell lines, a clear separation between the signals of NP-Chi-xPEI (red) and DNA (green) was observed (Figure 6b), which indicates the releasing of DNA from NP-Chi-xPEI. Although not all the DNA that arrived in the perinuclear region can successfully enter cell nuclei, indicating the nuclear envelope could be a hurdle for DNA delivery and transfection in this case, NP-Chi-xPEI potentially facilitated the nuclear uptake of DNA as the DNA found within cell nuclei were mostly accompanied by the presence of NP-Chi-xPEI in vicinity (indicated by white arrows in Figure 6b).

These observations demonstrated that NP-Chi-xPEI-DNA can be readily internalized by all three cell lines and effectively release its DNA payload intracellularly. Meanwhile, NP-Chi-xPEI could possibly enhance the nuclear uptake of DNA for facile transcription. It is also noted that the NP-Chi-xPEI-DNA achieved the highest cell uptake in SKBR3 cells followed by a slightly lower uptake in MCF7 and 4T1 cells. Regarding the nuclear entry of DNA, the highest amount of DNA found in cell nuclei were in SKBR3 cells. MCF7 also showed clear DNA nuclear entry. Although sufficient NP-Chi-xPEI-DNA was able to travel past 4T1’s plasma membrane, only a small fraction of NP-Chi-xPEI-DNA was able to enter 4T1 cell nuclei. This observation demonstrates that the cellular uptake and DNA release profile of NP-Chi-xPEI-DNA is cell-dependent, which is a common phenomenon observed for many other non-viral DNA carriers as well [63,64,65,66]. The fact that these three cell lines possess different sets of surface receptors may be one of the many factors that result in the cell-dependent performances of NP-Chi-xPEI-DNA.

### 3.4. In Vitro Biocompatibility of NP-Chi-xPEI

**Figure 7:**
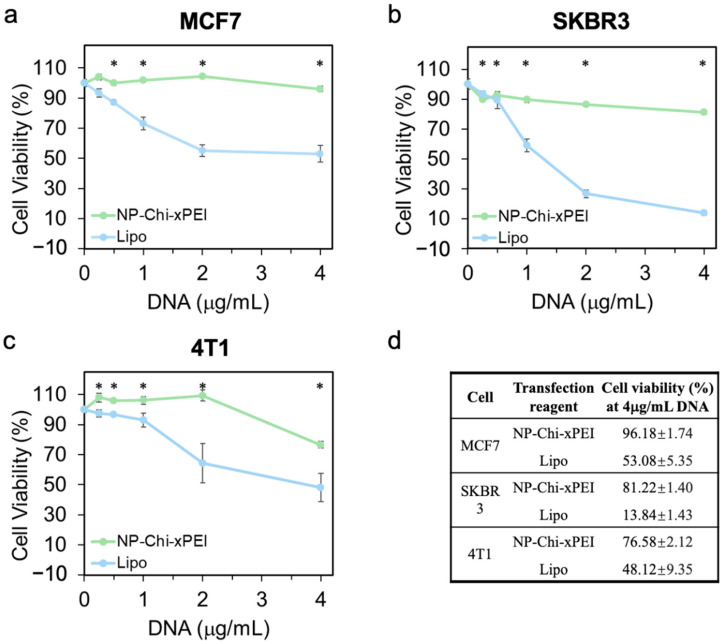
In vitro cell viability as a function of NP-Chi-xPEI-DNA/lipofectamine-DNA dose on three cancer cell lines. (**a**) MCF7, (**b**) SKBR3, and (**c**) 4T1 were treated with NP-Chi-xPEI-DNA (green) or lipofectamine-DNA (blue) at DNA concentration of 0.25, 0.5, 1, 2, 4 μg/mL for 48 h. (**d**) Summary of cell viability of NP-Chi-xPEI-DNA and lipofectamine-DNA treated cancer cells at 4 μg/mL for 48 h. * *p* < 0.05 between NP-Chi-xPEI and lipofectamine.

NP-Chi-xPEI-DNA’s biocompatibility on three breast cancer cell lines (MCF7, SKBR3, and 4T1) were assessed by treating these cell lines with NP-Chi-xPEI-DNA complex at various DNA doses (0.25, 0.5, 1, 2, 4 μg/mL) for 24 h. The cell groups were also treated with Lipofectamine 3000-DNA at the same DNA concentrations for comparison. Cell viability was quantified by the Alamar Blue assay (Figure 7). The results showed a general trend where cell viability decreased as the dose of DNA increased. At the highest dose of 4 μg/mL of DNA (Figure 7d), the viability of the NP-Chi-xPEI-DNA-treated cells were 96%, 81%, and 76%, whereas the viability of Lipofectamine 3000-treated cells were only 53%, 14%, and 48% for MCF7, SKBR3, and 4T1 cell lines, respectively. The mild cytotoxicity caused by NP-Chi-xPEI-DNA might be attributed to the cytotoxicity of PEI [67]. Nevertheless, the viability of the NP-Chi-xPEI-DNA-treated cells were significantly higher than that of the Lipofectamine 3000-treated cells at all dosages across all cell lines, demonstrating NP-Chi-xPEI-DNA’s superior biocompatibility to Lipofectamine 3000-DNA. The superior biocompatibility exhibited by NP-Chi-xPEI-DNA could be mostly attributed to the biodegradable Chi-xPEI as well as the small IONP core. Biodegradable disulfides in Chi-xPEI and the size conservation effect from IONP collectively prevent the presence of oversized DNA complexes that could otherwise carry too much positive charges and cause cell damages.

### 3.5. In Vitro Transfection Efficiency of NP-Chi-xPEI-DNA

**Figure 8:**
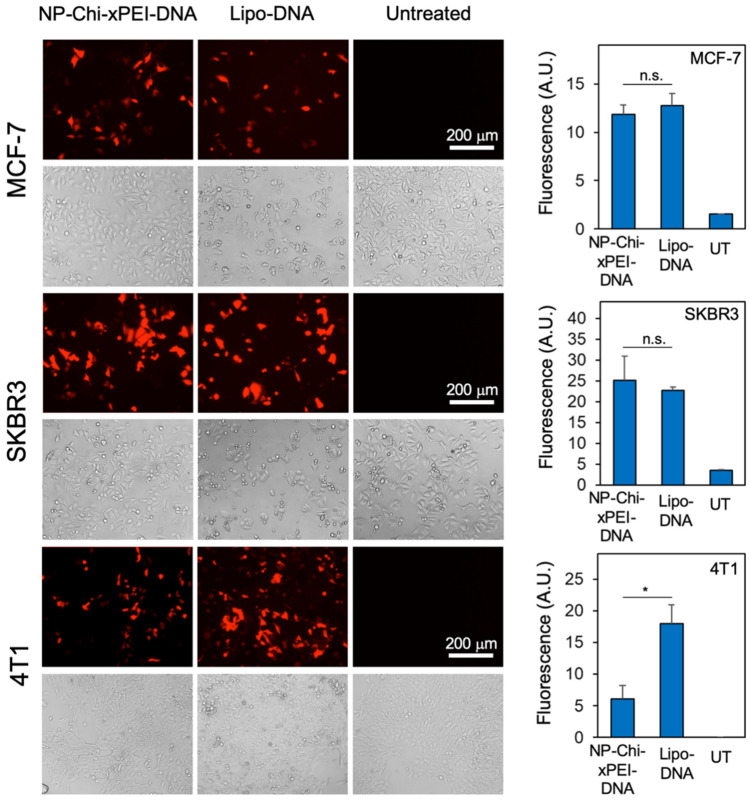
Fluorescent images of MCF7, SKBR3, and 4T1 breast cancer cells transfected by either NP-Chi-xPEI-DNA or lipofectamine-DNA (Lipo-DNA). The untreated cells served as the negative control group. Bright field images of the cells are also provided (directly below their corresponding fluorescent images). The quantitative transfection results of each cell line are presented in bar charts on the right side of the corresponding image panels. * *p* < 0.05, n.s. = statistically insignificant.

The transfection performance of NP-Chi-xPEI-DNA was evaluated on SKBR3, MCF7, and 4T1 breast cancer cell lines in vitro. Red fluorescence protein (RFP)-encoded pDsRed-MAX-N1 plasmid DNA was complexed with NP-Chi-xPEI and incubated for 30 min to form NP-Chi-xPEI-DNA complex before applying to breast cancer cell lines in vitro. The commercial transfection reagent Lipofectamine 3000 (Invitrogen) was used as the positive control in this experiment. The untreated cells served as the negative control group. From the transfection results, both NP-Chi-xPEI and lipofectamine 3000 showed various degrees of transfection when applied to the three breast cancer cell lines at the DNA concentration of 2 μg/mL (Figure 8). On SKBR3 and MCF7 cells, NP-Chi-xPEI-DNA was able to achieve a transfection efficiency comparable to, if not slightly higher than, that achieved by Lipofectamine 3000. NP-Chi-xPEI-DNA showed lower transfection efficiency than Lipofectamine 3000 on 4T1. In addition, NP-Chi-xPEI-DNA demonstrated higher transfection efficiency than Chi-xPEI-DNA (Figure S4). Notably, NP-Chi-xPEI-DNA enabled more viable cell growth and inflicted significantly lower cytotoxicity than Lipofectamine 3000 across all cell lines as exhibited by the bright field images: the NP-Chi-xPEI-DNA-treated cells showed similar proliferation and morphology to the untreated cells whereas Lipofectamine 3000 induced notable cell growth retardation and morphology alteration. This result conforms to the previous biocompatibility data. The untreated cell groups showed no detectable RFP signal, confirming that the breast cancer cells themselves did not produce autofluorescence to interfere with the RFP transfection results. The highly effective transfection and superior biocompatibility of NP-Chi-xPEI-DNA can be attributed to the combinatory effect of the small 10 nm IONP core and biodegradable Chi-xPEI that is made of nontoxic PEI with a small molecular weight of only 800 Da. IONP core and Chi-xPEI collectively confer the desirable physicochemical properties, serum stability, and biocompatibility to NP-Chi-xPEI-DNA system, which leads to a favorable intracellular trafficking profile and transfection efficiency in breast cancer cell lines in vitro.

The transfection performance of NP-Chi-xPEI-DNA showed a certain cell-type dependency, which could be explained by the previous cellular uptake and DNA release data (Figure 5). The amount of the cellular uptake of NP-Chi-xPEI-DNA and the amount of released DNA colocalized within cell nuclei were the highest in SKBR3 among all three cell lines, corresponding to the highest transfection efficiency of SKBR3. The amount of the cellular uptake of NP-Chi-xPEI-DNA and the amount of released DNA colocalized within cell nuclei were relatively lower in MCF7 cells than SKBR3 cells, which explains why MCF7 transfection was not as high. Although 4T1 cells also had a high amount of internalized NP-Chi-xPEI-DNA, few of the released DNA were found within the 4T1 cell nuclei, suggesting nuclear uptake could be the main obstacle for NP-Chi-xPEI-DNA transfection on 4T1 cells. These results collaboratively point out that cellular internalization, DNA release, and nuclear uptake all play important roles in the DNA transfection of cells. While the cell-type dependency can cause a variation in NP-Chi-xPEI-DNA’s transfection performance, the superior biocompatibility and high transfection efficiency across multiple breast cancer cell lines make NP-Chi-xPEI a safe and effective DNA carrier for breast cancer gene therapy.

## 4. Conclusions

We have presented a novel DNA carrier, NP-Chi-xPEI, that demonstrates the ability to deliver DNA to various breast cancer cells for DNA transfection in vitro. NP-Chi-xPEI has favorable physicochemical properties as a DNA carrier, including sub-hundred nm diameter, sufficient positive surface charges for DNA condensation, and protection and long-term serum stability. Although the cell uptake and DNA release profile of NP-Chi-xPEI-DNA is cell-dependent, NP-Chi-xPEI was able to successfully ferry DNA into different types of breast cancer cells and release DNA effectively for transfection. Through comparison studies with the commercially available transfection agent Lipofectamine 3000, NP-Chi-xPEI not only achieved high transfection efficiency but also displayed significantly lower cytotoxicity on multiple breast cancer cell lines. As safety must be the highest priority for a gene carrier in in vivo application, NP-Chi-xPEI may serve as an improved alternative to the current commercial gene carriers for breast cancer therapy.

## Figures and Tables

**Table 1:** Comparison of hydrodynamic size and zeta potential data between NP-Chi-xPEI-DNA and recently reported biodegradable PEI-based DNA carriers in literature.

Non-Viral DNA Carrier Material	Hydrodynamic Size	Zeta Potential	Reference
NP-Chi-xPEI-DNA	45.8 nm	18.3 mV	N/A
2,6-pyridinedicarboxaldehyde-Crosslinked 1.8k PEI	160–250 nm	17–27 mV	[29]
Disulfide-crosslinked 2k PEI modified with tyrosine	135.6 nm	62.1 mV	[31]
Disulfide-crosslinked 2.5k PEI	350–500 nm	20–40 mV	[32]
Disulfide and bisepoxide crosslinked 6k PEI	200 nm	20 mV	[34]
Oxidized glutathione crosslinked 0.6k PEI	200–400 nm	30 mV	[52]
Disulfide-crosslinked 1.8k PEI modified with cyclodextrin and poly-glutamic acid	250 nm	20 mV	[53]
Diglycidyl-1,2-cyclohexanedicarboxylate-crosslinked 10k PEI	125–201 nm	11–20 mV	[54]

**Table 2:** Comparison of hydrodynamic size and zeta potential data between NP-Chi-xPEI-DNA and recently reported IONP-based DNA carriers in literature.

Non-Viral DNA Carrier Material	Hydrodynamic Size	Zeta Potential	Reference
NP-Chi-xPEI-DNA	45.8 nm	18.3 mV	N/A
IONP-Catechol-Chitosan-25k PEI-DNA	54.3 nm	16.2 mV	[36]
IONP-Chondroitin-10k PEI-DNA	136 nm	15 mV	[37]
IONP-25k PEI-DNA	250 nm	19.2 mV	[38]
IONP-PAMAM Denrimer-DNA-25k PEI	190–285 nm	45 mV	[40]
IONP-1.8k PEI-DNA	50 nm (dry size)	10 mV	[41]
IONP covalently bound to DNA	241 nm	−26.4 mV	[55]
IONP-Lipids-DNA	50–100 nm	20 mV	[56]

## Data Availability

Data can be available upon request from the authors.

## Supplementary Materials

The following supporting information can be downloaded at: https://www.mdpi.com/article/10.3390/nano12040584/s1, Figure S1: Hydrodynamic size and zeta potential distribution profiles of Chi-xPEI-DNA; Figure S2: Gel electrophoresis image of Chi-xPEI-DNA; Figure S3: High resolution TEM micrograph of NP-Chi-xPEI-DNA; Figure S4: Fluorescence images of SKBR3 and 4T1 cells transfected with Chi-xPEI-DNA at DNA concentration of 2 µg/mL for 48 h.
