# Supplementary material for: Chitosan-Crosslinked Low Molecular Weight PEI-Conjugated Iron Oxide Nanoparticle for Safe and Effective DNA Delivery to Breast Cancer Cells
Source: Nanomaterials (Basel). 2022 Feb 9;12(4):584. doi: 10.3390/nano12040584 (PMC8876741; doi:10.3390/nano12040584)
Supplement: Supplementary file 1 [file nanomaterials-12-00584-s001.zip › nanomaterials-1558640-supplementary.pdf]

Supplementary Materials

Chitosan-Crosslinked Low Molecular Weight PEI-Conjugated Iron Oxide Nanoparticle for Safe and Effective DNA Delivery to Breast Cancer Cells

Guanyou Lin †, Jianxi Huang †, Mengyuan Zhang, Shanshan Chen and Miqin Zhang *

Department of Materials Science and Engineering, University of Washington, Seattle, WA 98195, USA; linguany@uw.edu (G.L.); jianxih@uw.edu (J.H.); zhangm28@uw.edu (M.Z.); schen268@jh.edu (S.C.)
* Correspondence: mzhang@uw.edu
† These authors contributed equally to this work.

The NP-Chi-xPEI-DNA complex was formed by mixing Chi-xPEI and DNA (pDsRed-MAX-N1 in 20mM HEPES buffer (pH 7.4) at Chi-xPEI:DNA wt/wt ratios of 40:1. Chi-xPEI-DNA solutions were incubated for at least 10 min with gentle rocking to allow formation of DNA complexes.

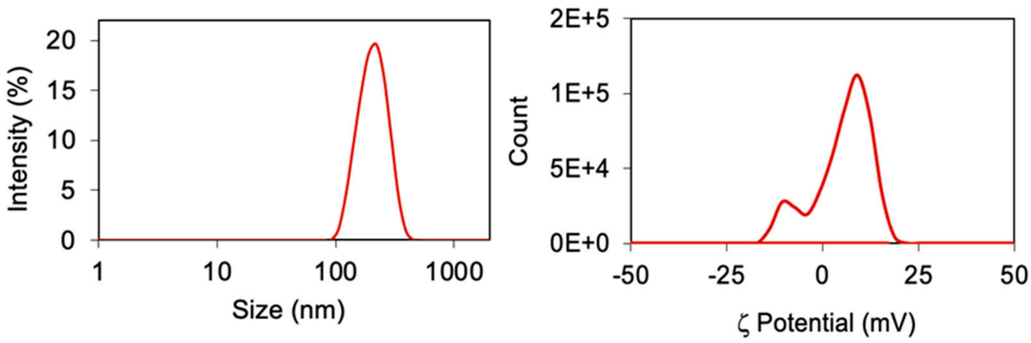

Figure S1. Hydrodynamic size and zeta potential distribution profiles of Chi-xPEI-DNA. The hydrodynamic size and zeta potential mean of Chi-xPEI-DNA are 223 nm and 18.7 mV respectively.

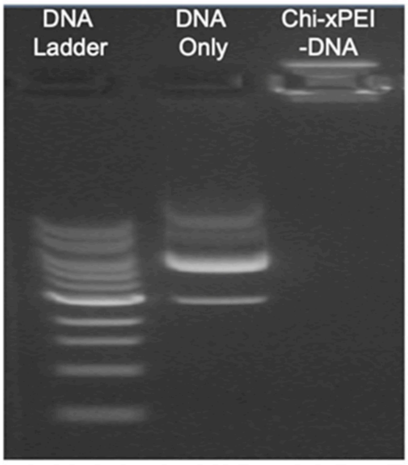

Figure S2. Gel electrophoresis image of Chi-xPEI-DNA. Although there wasn't free DNA band observed in the Chi-xPEI-DNA lane like that in the DNA ladder and the DNA only lanes, the free DNA signal is readily visible in the loading well.

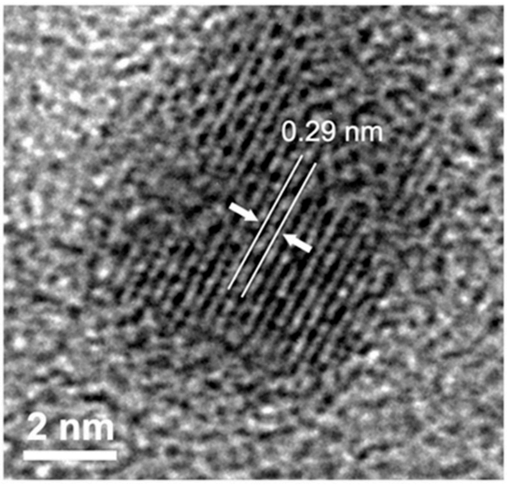

Figure S3. High resolution TEM micrograph of NP-Chi-xPEI-DNA. This image reveals the planar distance of 0.29 nm in NP crystal lattice, corresponding to the characteristic {220} planes of Fe_3O_4 crystalline.

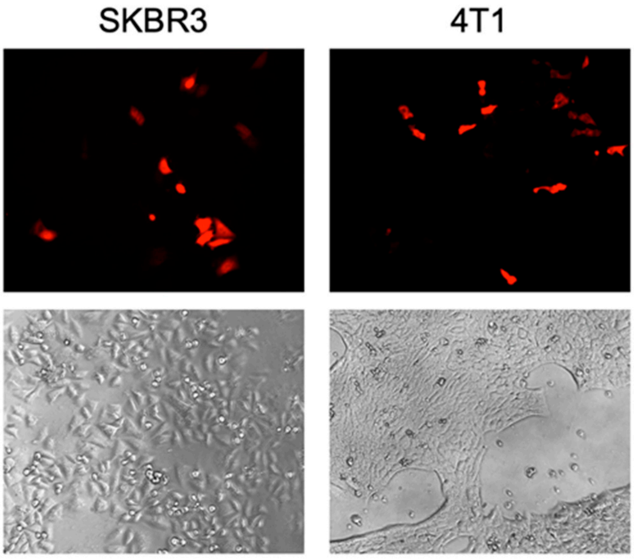

Figure S4. Fluorescence images of SKBR3 and 4T1 cells transfected with Chi-xPEI-DNA at DNA concentration of 2 μg/mL for 48 hours. The bottom row is bright field images corresponding to the fluorescence images in the top row.
